# USAG‐1 and Regenerative Dentistry, Therapeutic Implications and Future Directions: Review of the Literature

**DOI:** 10.1002/cre2.70301

**Published:** 2026-02-03

**Authors:** Zahra Moradi, Mohammadreza Karimi, Shirin Kolahdouz, Narges Arya, Vahid Akheshteh, Fatemeh Ayanzadeh, Yasmina Aalizadeh, Hanieh Moravvej, Marzie Keshavarzi, Amar Basri

**Affiliations:** ^1^ Memorial Dentistry for Kids Houston Texas USA; ^2^ School of Dentistry Bushehr University of Medical Sciences Bushehr Iran; ^3^ Oral and Dental Disease Research Center, School of Dentistry Shiraz University of Medical Sciences Shiraz Iran; ^4^ School of Dentistry Shahid Sadoughi University of Medical Sciences Yazd Iran; ^5^ Oral and Maxillofacial radiology, School of Dentistry Bushehr University of Medical Sciences Bushehr Iran; ^6^ Dr. Akheshteh Oral and Maxillofacial radiology Center Karaj Iran; ^7^ Department of Pediatric Dentistry, School of Dentistry Azad University (khorasgan Branch) Isfahan Iran

**Keywords:** bone morphogenetic protein (BMP) signaling, regenerative dentistry, tooth regeneration, USAG‐1, Wnt signaling pathway

## Abstract

**Objectives:**

Uterine Sensitization‐Associated Gene 1 (USAG‐1) is a bone morphogenetic protein (BMP) antagonist vital for tooth regeneration that is expressed in kidney, gingiva, and dental tissues.

**Material and methods:**

We analyzed recent studies focusing on USAG‐1 and its involvement in BMP and Wnt signaling pathways related to dental tissue repair and regeneration. Preclinical models and clinical trial data were examined to evaluate the efficacy of USAG‐1 inhibition as a therapeutic strategy. In addition, publicly available single‐cell RNA sequencing and STRING databases were analyzed to investigate the gene expression of *USAG‐1* in human tissues and its protein interactions, respectively.

**Results:**

RNA‐seq analysis confirmed that *USAG‐1* is expressed in a subset of secretory cell types in kidney, jaw, and gingiva that are important for cell growth and morphogenesis. Recent studies have also demonstrated that inhibiting USAG‐1 facilitates tooth regeneration by activating BMP‐mediated morphogenesis and improving outcomes in preclinical models. Engineered monoclonal antibodies that target USAG‐1 have shown that blocking the protein product of this gene can promote third dentition and alleviate congenital tooth agenesis. Clinical trials utilizing this antibody are currently underway, with prospects for commercial applications within the next decade. Despite these advancements, challenges related to safety, specificity, and delivery mechanisms remain.

**Conclusions:**

This review underscores the transformative potential of USAG‐1‐based therapies in regenerative dentistry, offering a paradigm shift in dental care by enabling biologically authentic tooth regeneration. However, the realization of these advancements in clinical practice requires overcoming significant barriers, including ensuring safety, optimizing delivery systems, and addressing ethical concerns. Continued interdisciplinary research is essential to fully harness the potential of USAG‐1 in regenerative dentistry.

## 1 Introduction

*Uterine Sensitization‐Associated Gene 1* (*USAG‐1*), also known as human *SOSTDC1*, has emerged as a significant focus in regenerative medicine, dentistry, and renal research due to its role as an antagonist of bone morphogenetic protein (BMP) and Wnt signaling. *USAG‐1* was first discovered to be expressed in the rat uterus, but has since been found to be expressed in the kidney, salivary glands, and dental tissues during embryogenesis and into adult stages, highlighting its potential dual function throughout development (Yanagita 2005). Its expression in renal tubules suggests a regulatory role in protecting against renal damage, as evidenced by studies demonstrating that USAG‐1 can inhibit BMP‐7, a protein known for its protective properties in the kidney (Kiso et al. 2014). In dental contexts, USAG‐1 has garnered interest for its possible implications in tooth regeneration. As adults, humans lose the ability to regenerate teeth and therefore any dental damage that is incurred is typically irreversible (Murashima‐Suginami et al. 2021).

This has spurred ongoing research into therapeutic interventions aimed at promoting tooth regrowth. An important aspect of this research is the modulation of USAG‐1 activity, which may open avenues for enhancing BMP signaling in dental tissues, potentially leading to advancements in regenerative dental therapies (Murashima‐Suginami et al. 2008). Recent findings indicate that USAG‐1 also influences the Wnt signaling pathway, which is vital for various developmental processes, including those related to tooth and bone formation. The interplay between BMP and Wnt pathways presents an intriguing area of exploration, particularly regarding the enhancement of dental tissue repair and regeneration. With USAG‐1‐focused clinical trials for tooth regrowth treatments already underway, it is critical that we understand the mechanisms behind these potential therapies and their effectiveness in restoring dental health (Takahashi et al. 2024).

USAG‐1 plays a pivotal regulatory role in tooth development by functioning as a dual antagonist of the BMP and Wnt signaling pathways, both of which are central to dental morphogenesis and regeneration. BMP signaling governs the differentiation of dental stem cells and the formation of enamel, dentin, and pulp, whereas Wnt signaling directs cell proliferation, patterning, and the initiation of tooth buds. Through its inhibitory actions on these pathways, USAG‐1 helps ensure balanced tissue development and prevents aberrant mineralization. Experimental studies have shown that loss or inhibition of USAG‐1 can induce supernumerary teeth and reactivate aspects of the third dentition, highlighting its importance in postnatal regeneration. Furthermore, USAG‐1 interacts with key regulatory proteins such as LRP5, DKK4, BMP2, BMP4, and BMP7, integrating into broader molecular networks that coordinate dental tissue growth and homeostasis. These mechanistic insights underscore why USAG‐1 is considered a promising therapeutic target for regenerative dentistry, with early‐stage preclinical work and ongoing clinical trials exploring monoclonal antibodies aimed at reactivating tooth formation in adults (Murashima‐Suginami et al. 2008; Takahashi et al. 2024; Murashima‐Suginami et al. 2007).

While several reviews have discussed the biological functions of USAG‐1 in tooth development and regeneration, we integrate a review of the pertinent literature with single‐cell RNA sequencing data to uncover cell type specificity (including fibroblast and basal cell populations) of USAG‐1 expression in human gingiva. Further, we combine transcriptomic findings with protein–protein interaction analyses to highlight how USAG‐1 intersects with key regulatory nodes such as LRP5, DKK4, BMP2, BMP4, and BMP7 (Szklarczyk et al. 2023). This integrative perspective not only advances the mechanistic understanding of USAG‐1 but also frames its potential applications in personalized regenerative dental therapies.

## 2 Methods

### 2.1 Review Inclusion Criteria

A thorough review of the literature was conducted to identify relevant studies on USAG‐1 and its role in regenerative dentistry. Searches were performed in PubMed, Scopus, and Web of Science databases, covering publications from January 2000 to June 2025. The search string and its variations were used: “USAG‐1” OR “uterine sensitization‐associated gene‐1” AND “tooth regeneration” OR “regenerative dentistry.”

Boolean operators and database‐specific filters (language and article type) were applied as appropriate. All retrieved records were imported into a reference manager, and two independent reviewers screened titles and abstracts for relevance. Inclusion criteria encompassed original research articles, review papers, and clinical studies focusing on USAG‐1 function, molecular mechanisms, preclinical models, or therapeutic applications. Exclusion criteria included non‐English publications, conference abstracts without full text, and studies unrelated to USAG‐1 or dental regeneration. Discrepancies during screening were resolved through discussion or consultation with a third reviewer.

### 2.2 RNA Expression Analysis Using DISCO

RNA expression levels and patterns of USAG‐1 were assessed using DISCO (https://disco.bii.a-star.edu.sg/genepage/SOSTDC1) (Li et al. 2022). For analysis, the gene of interest (USAG‐1) was queried in DISCO, and the gingiva tissue atlas was selected. Specific cell types expressing USAG‐1 were identified, and expression metrics were extracted.

**Figure 1:**
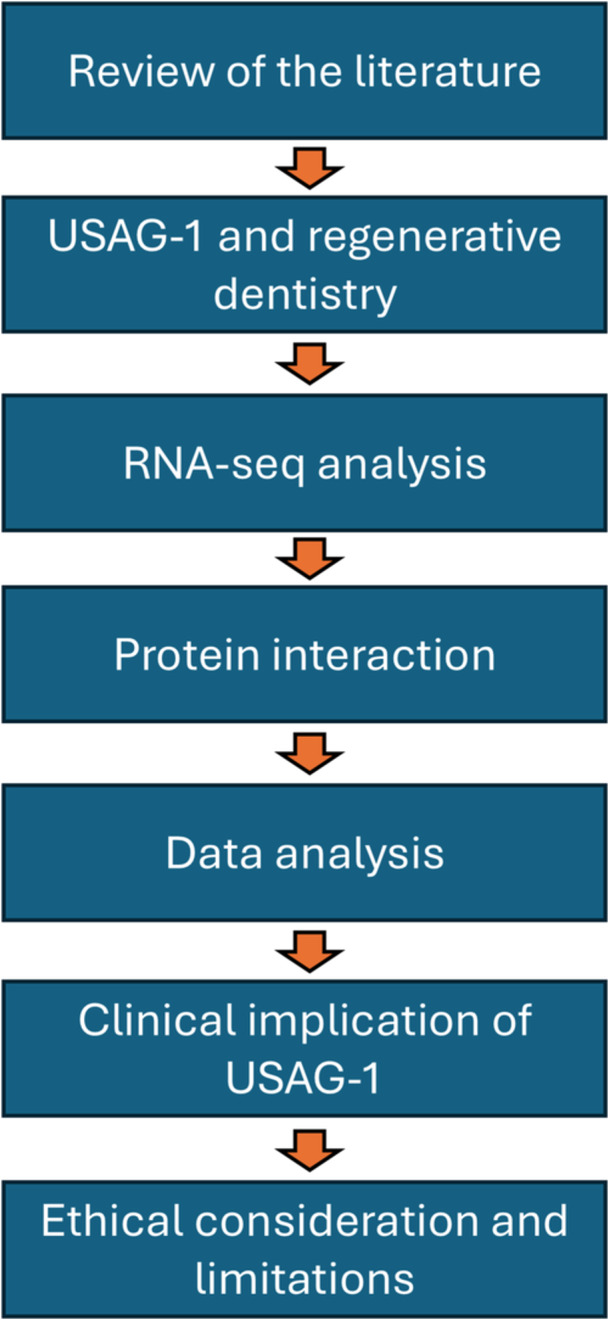
A PRISMA flowchart summarizing study selection. USAG‐1, Uterine Sensitization‐Associated Gene 1.

The following metrics were recorded for each cell type: Gene: gene symbol analyzed, Atlas: dataset/tissue source (e.g., gingiva), Cell Type: all specific cell population (e.g., fibroblasts, basal cells), LogFC: log fold change between two comparison groups, Adjusted p‐value: statistical significance after multiple testing correction, Group 1/Group 2: conditions being compared, Percentage 1/Percentage 2: fraction of cells expressing the gene in Group 1 and Group 2, and Type: type of analysis (e.g., Cell Type DEG). These data are provided in Supporting Information: Table S1. Default DISCO pipelines were used for preprocessing, normalization, and filtering according to the web tool's specifications. Expression thresholds were determined based on the proportion of cells expressing the gene above the default detection limit in DISCO (Li et al. 2022). We also recruited STRING (Szklarczyk et al. 2023) database to study the interaction of the proteins Figure 1. Details of the interaction metrics is summarized in Supporting Information: Table S2.

DISCO database details: Version: DISCO v2.4 (released February 2024); Access date: 15 January 2025; QC thresholds: Cells with < 200 detected genes removed, Cells with > 5000 detected genes removed, Mitochondrial gene content > 20% removed. Normalization: Log‐normalized counts using Seurat default (“LogNormalize”, scale factor = 10,000). Expression detection threshold: ≥ 0.1 normalized expression (DISCO default) and ≥ 1% of cells expressing USAG‐1 in a cluster.

STRING database details: Version: STRING v12.0 (released 2023), Access date: 20 January 2025, Confidence score cutoff: 0.70 (high confidence), Active interaction sources: experiments + curated databases + co‐expression, No text mining sources used (deactivated for higher biological specificity).

RNA‐seq preprocessing: We clarified that DISCO's built‐in preprocessing pipeline was used (SCTransform normalization disabled; LogNormalize applied instead). Cluster identification performed using 30 principal components and Leiden clustering (resolution = 0.8).

For DISCO, all values (logFC, adjusted p‐values, cluster percentages, expression thresholds) were exported directly from the platform using identical filters and QC settings.

For STRING, all interaction scores, confidence values, and enrichment statistics were extracted using a fixed confidence cutoff (0.700) and the same active/inactive interaction sources.

For literature‐derived variables, numerical data (sample sizes, effect measures, signaling outcomes, and regenerative endpoints) were recorded using a single standardized form, reviewed independently by two authors.

## 3 Results

The exploration of USAG‐1 inhibition as a viable approach for tooth regeneration has gained traction in recent years. Comprehensive meta‐analyses indicate that inactivating USAG‐1 not only addresses congenital tooth agenesis but also facilitates late‐stage tooth morphogenesis and activates third dentition in experimental models (Togo et al. 2016). The potential applications of this research are significant, particularly in regenerative medicine, as the proposed monoclonal antibody therapeutic aims to treat genetic tooth loss and deformation by promoting tooth regeneration in adults. Since 2021, research conducted on mouse models has paved the way for the development of this therapy (Mishima et al. 2021), with active clinical trials involving adult subjects at Kyoto University Hospital beginning in 2024 (Takahashi et al. 2024). The anticipated timeline for commercial availability of this therapy is projected for 2030, contingent upon successful clinical outcomes demonstrating both efficacy and safety. However, the complexity of human dental variability presents challenges that researchers must navigate before this therapy can become widely utilized. A key focus of ongoing studies is determining the reliability of USAG‐1 inhibition to regenerate teeth across different individuals and to adequately address the unique structures of various dental forms, such as molars and incisors (Gašparovič et al. 2024).

Furthermore, ethical considerations regarding accessibility and the implications of such regenerative treatments on dental education and practice must be addressed to ensure responsible application of USAG‐1‐based therapies. Our meta‐analysis also highlights several obstacles that remain before the clinical application of USAG‐1 inhibition can be fully realized. These challenges include ensuring the safety and specificity of treatment, developing efficient delivery mechanisms, and navigating regulatory approval processes. Preliminary findings from animal models have shown minimal side effects; however, extensive human trials are necessary to evaluate potential impacts on other tissues where potentially impacted signaling pathways are active (Takahashi et al. 2024).

### 3.1 Role of USAG‐1 in Tooth Regeneration

Recent studies have established the important role for USAG‐1 in tooth regeneration, with studies highlighting its antagonistic effects on critical signaling pathways involved in dental development. USAG‐1 inhibits bone morphogenetic protein (BMP) and Wnt signaling, both essential for the differentiation of dental stem cells, enamel, dentin, and pulp formation. By modulating these pathways, USAG‐1 effectively prevents the regeneration of teeth post‐development, making it a key target for regenerative therapies (Poorani et al. 2022).

### 3.2 Preclinical and Clinical Research Outcomes

Meta‐analyses of preclinical studies involving mouse models indicate that the inhibition of USAG‐1 can promote tooth regeneration and address conditions such as congenital tooth agenesis. Studies have shown that using monoclonal antibodies against USAG‐1 results in the successful regrowth of functional teeth in treated mice, with normal anatomical structures, including enamel and dentin, being formed. Furthermore, the regenerated teeth demonstrated biological integration with the surrounding jawbone, ensuring stability and proper alignment with adjacent teeth (Ravi et al. 2023).

### 3.3 Exploring USAG‐1 Expression Using Single‐Cell RNA Sequencing

**Figure 2:**
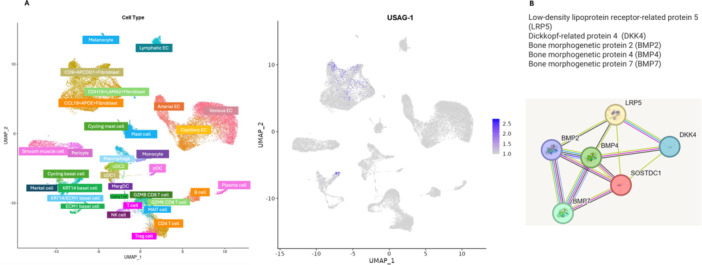
(A) USAG‐1 is expressed in a subset of fibroblasts in the gingiva that are crucial for tissue regeneration. (A) (left): Cell type clusters based on RNA‐sequencing data. Panel A (right): Blue dots are representative of the cells that expresses USAG‐1. (B) USAG‐1 interacts with proteins that are important for bone regeneration. USAG‐1, Uterine Sensitization‐Associated Gene 1.

Based on RNA sequencing data, USAG‐1 is exclusively expressed in a subset of fibroblasts and basal cells of gingiva. These cell types include CD9+APCDD1+ fibroblasts, CDH19^+^LAMA2^+^ fibroblasts, and Krt14 basal cells. CD9 and APCDD1 are cell surface markers of human dermal fibroblasts. Interestingly, CD9 has been implicated in promoting the transition of fibroblasts to myofibroblasts under hypoxic conditions. This process is important for wound healing and tissue remodeling. Furthermore, APCDD1 is known to be highly expressed in papillary fibroblasts. APCDD1 can modulate the Wnt signaling pathway and regulate the odontoblastic differentiation of dental pulp stem cells (Figure 2A) (Li et al. 2022; Fadl and Leask 2023). Therefore, these human gingival fibroblasts (HGFs) have demonstrated significant capabilities in regenerating both hard and soft dental and periodontal tissues, highlighting their potential in dental regenerative therapies (Lu et al. 2019). Additionally, Cadherin‐19 (CDH19) is a type II cadherin involved in calcium‐dependent cell‐cell adhesion, primarily expressed in neural crest cells and Schwann cell precursors. Laminin subunit alpha‐2 (LAMA2) is a component of the extracellular matrix, playing a crucial role in the structural scaffolding of tissues. Both CDH19 and LAMA2 are important in cellular adhesion and extracellular matrix composition. These processes are crucial for tissue development and regeneration, suggesting that CDH19 + LAMA2+ fibroblast populations potentially influence tissue regeneration in the gingiva. Moreover, studies on dermal fibroblast dedifferentiation have demonstrated that specific treatments can induce dermis‐derived cells to participate in multiple cell lineages during limb regeneration (Satoh et al. 2022).

Keratin 14 (Krt14) is a protein predominantly expressed in basal epithelial cells, including those in the dental epithelium during tooth development. Studies have shown that Krt14, along with other keratins like Krt5 and Krt17, is robustly expressed in the dental epithelium during various developmental stages, indicating a role in maintaining the structural integrity and function of these cells (Inada et al. 2024). Additionally, research on epithelial stem cells in tooth and taste bud development indicates that the oral epithelium retains inherent plasticity to form tooth and taste‐like cell types. This plasticity is mediated by bone morphogenetic protein (BMP) signaling, which influences the fate of Krt14‐positive basal cells during development (Bloomquist et al. 2019). Single‐cell transcriptomic analyses reveal distinct cell populations expressing USAG‐1 in gingival tissues, as summarized in Table 1. In addition, Table 2 provides an integrative summary of USAG‐1 expression patterns, interventions, and regenerative outcomes across preclinical, clinical, and single‐cell transcriptomic studies. These findings suggest that these fibroblasts play a significant role in tissue regeneration.

**Table 1:** Single‐cell transcriptomic analyses reveal distinct cell populations expressing USAG‐1 in gingival tissues.

Cell type	Markers	Function in dental regeneration
Fibroblast	CD9, APCDD1	Tissue remodeling, Wnt pathway modulation
Fibroblast	CDH19, LAMA2	Extracellular matrix composition, tissue regeneration
Basal epithelial cells	Krt14	Maintaining dental epithelial structure

Abbreviation: USAG‐1, Uterine Sensitization‐Associated Gene 1.

**Table 2:** Summary of USAG‐1 expression and its effects on tooth regeneration.

Study	Model/cell type	USAG‐1 expression frequency	Intervention	Effect size/outcome
Murashima‐Suginami et al. (2021)	Mouse	Not reported (preclinical model)	Anti‐USAG‐1 monoclonal antibody	100% of treated mice showed functional tooth regeneration; normal enamel/dentin
Kyoto University Hospital, 2024	Adult humans (Phase I)	Not reported	Anti‐USAG‐1 monoclonal antibody	Safety/tolerability assessed; preliminary efficacy observed
Human gingival fibroblasts	CD9+APCDD1+	~45%–50% of fibroblasts	N/A (expression data)	Promotes tissue remodeling, Wnt pathway modulation
Human gingival fibroblasts	CDH19+LAMA2+	~30%–35% of fibroblasts	N/A	Supports extracellular matrix composition, tissue regeneration
Basal epithelial cells	Krt14+	~60% of basal epithelial population	N/A	Maintains dental epithelial structure, supports regenerative plasticity

*Note:* Expression frequencies are based on single‐cell RNA‐seq data; percentages reflect proportion of specific cell subsets expressing USAG‐1 markers. Effect sizes in preclinical studies are represented qualitatively where numerical values were not reported.

Abbreviation: USAG‐1, Uterine Sensitization‐Associated Gene 1.

### 3.4 USAG‐1 Interactions

USAG‐1 is a dual inhibitor of both the Wnt and BMP signaling pathways. By modulating the activity of several important targets including Low‐Density Lipoprotein Receptor‐Related Protein 5 (LRP5) and BMP proteins (like BMP2, BMP4, and BMP7), USAG‐1 ensures that dental and bone tissue growth remains balanced, preventing excessive mineralization or abnormal development. Several key signaling proteins contribute to dental tissue regeneration by regulating cell differentiation, bone formation, and developmental patterning (Szklarczyk et al. 2023).

LRP5 functions as a coreceptor in the Wnt signaling pathway, working alongside frizzled receptors to mediate Wnt protein signals. This pathway is essential for determining cell fate and maintaining self‐renewal during embryonic development and tissue repair, including in tooth and bone regeneration. LRP5 is especially involved in regulating the proliferation and specialization of osteoblasts, the cells responsible for bone formation. DKK4 (Dickkopf‐Related Protein 4) acts as a Wnt signaling inhibitor. It prevents Wnt proteins from binding to LRP5/6 by forming a complex with KREMEN, leading to the internalization and removal of the receptors from the cell surface. This inhibition fine‐tunes Wnt activity during crucial developmental processes including tooth morphogenesis, limb formation, and somitogenesis. In adults, DKK4 continues to influence bone formation and may be involved in bone‐related diseases. BMP2 is a powerful inducer of bone and cartilage formation, including dental hard tissues. It promotes the transformation of muscle precursor cells into osteoblasts through the EIF2AK3–EIF2A–ATF4 signaling pathway, ultimately leading to the expression of genes critical for bone development. BMP2 also stimulates TMEM119, which enhances ATF4 expression, further supporting osteogenic differentiation. BMP4, like BMP2, drives cartilage and bone formation, but it also has distinct roles in tooth development, mesoderm induction, and tissue repair. It interacts with other factors like PTHLH to regulate the formation of structures like mammary ducts and hair follicles, showing its broad developmental functions. In addition, BMP7 supports epithelial osteogenesis, a process important in dental tissue formation, and plays a role in maintaining calcium balance and overall bone homeostasis (Szklarczyk et al. 2023) (Figure 2B). Hence, USAG‐1 modulates important targets that are crucial for bone regeneration.

Beyond BMP and Wnt signaling, emerging evidence suggests that USAG‐1 may also influence downstream signaling cascades, particularly the SMAD pathway, which mediates transcriptional responses to BMP activation and regulates odontoblast differentiation. Additionally, crosstalk between BMP/Wnt signaling and the MAPK/ERK pathway has been reported in tooth morphogenesis and bone formation, indicating that USAG‐1's regulatory effects may extend to broader intracellular signaling networks. While these pathways are less well characterized in the context of USAG‐1, their involvement here highlights the multifaceted molecular environment in which USAG‐1 operates (Kiso et al. 2014; Omi et al. 2020; Li et al. 2021). The multi‐faceted mechanisms of USAG‐1 regulation of BMP/Wnt signaling is summarized in Table 3.

**Table 3:** USAG‐1 exerts its regulatory function through interactions with multiple signaling mediators.

Target	Pathway	Role in regeneration
LRP5	Wnt	Osteoblast proliferation
DKK4	Wnt	Fine‐tunes Wnt signaling
BMP2	BMP	Induces dental hard tissue formation
BMP4	BMP	Tooth development and repair
BMP7	BMP	Epithelial osteogenesis, bone homeostasis

Abbreviations: BMP, Bone Morphogenetic Protein; DKK4, Dickkopf‐Related Protein 4; LRP5, Low‐Density Lipoprotein Receptor‐Related Protein 5; USAG‐1, Uterine Sensitization‐Associated Gene 1.

### 3.5 Therapeutic Implications and Future Directions

As USAG‐1 research progresses, clinical trials have begun to evaluate the safety and efficacy of monoclonal antibody treatments aimed at blocking USAG‐1 function. Initial trials are focused on adults who have lost teeth due to various reasons, including genetic factors, while future studies will expand to include children and older adults with different dental deficiencies. The expectation is that these treatments could not only restore lost teeth but also improve overall dental health and function, reducing the reliance on traditional dental interventions such as dentures and implants. The current findings underscore the therapeutic potential of USAG‐1 inhibition in regenerative dentistry and bone disorders. Targeting USAG‐1 could lead to innovative treatments for tooth loss, offering a minimally invasive and biologically authentic solution. Ongoing clinical trials, such as those initiated at Kyoto University Hospital, aim to assess the safety and effectiveness of USAG‐1 monoclonal antibodies in adult subjects, with the goal of eventual commercialization within the next decade (Murashima‐Suginami et al. 2021; Takahashi et al. 2024).

In terms of clinical translation, the development of monoclonal antibodies targeting USAG‐1 is progressing along a structured pathway from preclinical validation to human application. Preclinical studies in mice demonstrated that USAG‐1 inhibition can stimulate the regeneration of anatomically complete and functional teeth, laying the foundation for first‐in‐human investigations. Although the clinical trial has not yet been published in any peer‐reviewed journal, media has announced that a Phase I trial initiated at Kyoto University Hospital in 2024 is currently enrolling adult participants with acquired tooth loss to evaluate safety, tolerability, and preliminary efficacy. The anticipated transition to Phase II studies by 2026 will focus on individuals with congenital tooth agenesis, with primary endpoints including the induction of new tooth structures, integration with surrounding bone, and assessment of long‐term durability. A subsequent Phase III program will likely evaluate broader populations across different age groups and dental conditions, with commercialization projected for 2030, pending regulatory approval (Murashima‐Suginami et al. 2021; Takahashi et al. 2024; Ravi et al. 2023; Verma et al. 2025).

Together, these data emphasize both the remarkable progress achieved and the critical need for rigorous long‐term evaluation before widespread clinical adoption.

### 3.6 Broader Impact on Dental Care

The potential for a successful USAG‐1 targeting therapy could revolutionize dental care by providing a more natural solution for tooth loss. Unlike current methods that merely replace missing teeth, this approach aims to regenerate natural teeth, addressing both esthetic and functional concerns. This could significantly improve the quality of life for many individuals suffering from dental issues, particularly those with congenital conditions that limit tooth development. The discovery of the genetic causes related to tooth development, particularly the role of the *USAG‐1* gene, has significant implications for regenerative medicine and dentistry. Research indicates that inhibiting USAG‐1 can enhance tooth growth by increasing the activity of Bone Morphogenetic Protein (BMP), which is essential for tooth formation. This finding suggests that targeted therapies could potentially address congenital tooth agenesis and promote tooth regeneration in adults (Sadrabad et al. 2024).

### 3.7 Challenges and Ethical Considerations

Despite promising findings, challenges remain in translating USAG‐1‐targeted therapies for tooth regeneration to widespread clinical practice. Critical issues include safety, specificity, and effective delivery mechanisms of USAG‐1 inhibitors, alongside ethical and regulatory considerations. These therapies raise important questions regarding accessibility, the potential transformation of dental practice, and equitable implementation, particularly in low‐resource settings. Addressing these concerns will be essential for the responsible development and application of USAG‐1‐based therapies, ensuring that advancements benefit a broad population while maintaining safety, efficacy, and fair global access (Takahashi et al. 2024; Sadrabad et al. 2024).

#### 3.7.1 Regulatory Hurdles

The regulatory landscape for regenerative therapies varies globally, with many countries lacking comprehensive frameworks for cell, tissue, and gene therapy products. The World Health Organization (WHO) has emphasized the need for national regulatory authorities to establish and harmonize regulation for such therapies to ensure safety, efficacy, and quality. In the United States, the Food and Drug Administration (FDA) has developed a framework for regulating regenerative medicine, including gene therapies and monoclonal antibodies. However, the complexity of these therapies and the rapid pace of innovation pose challenges in keeping regulatory processes current and effective (Sanduja et al. 2023; Celis 2010; McGrath et al. 2024).

#### 3.7.2 Cost and Accessibility

The high cost of developing and administering monoclonal antibody therapies raises concerns about affordability and accessibility, particularly in low‐resource settings. These therapies often involve complex manufacturing processes and require specialized healthcare infrastructure, which may not be available in underserved regions (Takahashi et al. 2024; Celis 2010; Takahashi et al. 2020).

#### 3.7.3 Global Access and Equity

Ensuring global access to regenerative therapies necessitates international collaboration and the establishment of equitable distribution systems. The WHO's Expert Advisory Committee on Developing Global Standards for Governance and Oversight of Human Genome Editing has called for consistent worldwide standards to guide ethical applications of genome editing technologies, which are integral to regenerative medicine. Ethical considerations also extend to the informed consent process, ensuring that patients fully understand the potential risks and benefits of participating in clinical trials or by receiving treatments. This is particularly important in low‐resource settings, where patients may have limited access to information and healthcare services (Murashima‐Suginami et al. 2021; Takahashi et al. 2024; Mishima et al. 2021; Celis 2010; McGrath et al. 2024; Emanuel 2000).

## 4 Discussion

The role of USAG‐1 as a regulator of BMP and Wnt signaling has opened new avenues in regenerative dentistry, offering promising therapeutic possibilities for tooth regeneration and congenital dental conditions. Current research underscores the transformative potential of USAG‐1 inhibition to revolutionize dental care, providing biologically authentic alternatives to conventional replacements like implants and dentures. While preclinical and initial clinical findings are encouraging, the translation of these therapies to widespread clinical practice still requires overcoming significant challenges. These include ensuring the safety and specificity of USAG‐1‐targeted treatments, addressing the variability of human dental anatomy, and developing efficient delivery mechanisms. A summary of key preclinical and early clinical investigations exploring anti‐USAG‐1 therapies is provided in Table 4.

**Table 4:** Preclinical and clinical USAG‐1 studies.

Study	Model	Intervention	Outcome
Murashima‐Suginami et al. (2021)	Mouse	Anti‐USAG‐1 monoclonal antibody	Functional tooth regeneration; normal enamel/dentin
Kyoto University Hospital, 2024	Adult humans (Phase I)	Anti‐USAG‐1 monoclonal antibody	Safety/tolerability, preliminary efficacy

Abbreviation: USAG‐1, Uterine Sensitization‐Associated Gene 1.

Tooth regeneration is a multifaceted process encompassing the coordinated regeneration of dentin, pulp, gingival tissues, and alveolar bone. Current evidence suggests that USAG‐1 inhibition most directly influences dentin and pulp regeneration, primarily through the enhancement of BMP2, BMP4, and BMP7 signaling, which drives odontoblast differentiation and reparative dentin formation. In parallel, modulation of canonical Wnt signaling affects gingival fibroblast activity and epithelial proliferation, processes essential for gingival repair and regeneration. Moreover, BMP–Wnt crosstalk downstream of USAG‐1 may indirectly support osteoblast differentiation and alveolar bone remodeling, highlighting its broader regenerative potential. By linking USAG‐1 activity to these distinct regenerative components, our review underscores this gene's multifaceted therapeutic promise in dental tissue regeneration (Kiso et al. 2014; Omi et al. 2020; Li et al. 2021).

Despite this promise, the ethical implications of accessibility and equitable distribution must be addressed to ensure that these advancements benefit a broad population. As clinical trials advance and therapeutic options near commercialization, the integration of USAG‐1‐based therapies into dental practice could mark a paradigm shift, enhancing quality of life for individuals affected by dental diseases or tooth loss. Continued interdisciplinary research and collaboration will be pivotal in overcoming barriers and realizing the full potential of USAG‐1 in regenerative dentistry (Murashima‐Suginami et al. 2007; Verma et al. 2025).

Here, we have integrated recent single‐cell RNA sequencing data together with established knowledge of USAG‐1 biology, offering novel insights into this important therapeutic stragegy. By mapping USAG‐1 expression to distinct gingival fibroblast subtypes (e.g., CD9⁺APCDD1⁺, CDH19⁺LAMA2⁺) and basal epithelial cells (Krt14⁺), we highlight previously underappreciated cell populations that may play critical roles in dental regeneration. This cellular‐level perspective provides a framework for developing personalized regenerative strategies, since heterogeneity among fibroblast and basal cell populations can influence therapeutic outcomes. Moreover, by contextualizing USAG‐1 interactions with key signaling mediators such as LRP5, DKK4, BMP2, BMP4, and BMP7, this review uniquely emphasizes the multifactorial regulatory network underpinning tooth development and regeneration. Taken together, our work proposes a novel mechanistic model and cell‐type‐specific understanding of USAG‐1, thereby bridging molecular biology with translational opportunities in precision dental medicine (Li et al. 2022, 2025).

### 4.1 Study Limitations

While current evidence underscores the promising potential of USAG‐1–targeted therapies for tooth regeneration, several important limitations must be acknowledged. First, much of the available data is derived from preclinical animal models, and the translation of these findings to humans remains uncertain due to potential variability in human dental anatomy and biological responses. However, such models have been utilized in many instances with great clinical success, demonstrating the high degree of conservation between models and human biology. Second, delivery mechanisms for USAG‐1 inhibitors, including monoclonal antibodies, are still under development, and their efficiency, specificity, and long‐term safety in humans have yet to be fully established. Much of this knowledge may be born out in existing clinical trials. Third, the influence of patient‐specific factors—such as age, genetic background, and underlying health conditions—on treatment efficacy is not yet well understood. This is arguably true for many current therapies and will likely be explored systematically upon positive safety and efficacy findings in clinical trials. Finally, economic and infrastructural barriers, particularly in low‐resource settings, may limit the widespread clinical application of these therapies. This is a common issue for a large number of existing therapeutics and is not particularly unique to this methodology. Solutions that address these issues requires significant investment into healthcare infrastructure by government and other interested entities. Altogether, these gaps need to be continued to be addressed through additional preclinical research, optimization of delivery strategies, and well‐designed clinical trials will be essential to fully realize the therapeutic potential of USAG‐1–based regenerative interventions (Takahashi et al. 2024; Togo et al. 2016; Mishima et al. 2021; Ravi et al. 2023; Satoh et al. 2022). The projected clinical development pathway for USAG‐1–targeted therapies is summarized in Table 5.

**Table 5:** Clinical trial timeline for USAG‐1 therapy.

Phase	Population	Year	Endpoint
Phase I	Adults with acquired tooth loss	2024	Safety, tolerability, preliminary efficacy
Phase II	Congenital tooth agenesis	2026	Tooth induction, integration with bone
Phase III	Broader populations	2028+	Long‐term durability, functional outcomes
Commercialization	General population	2030	Pending regulatory approval

## 5 Conclusion

USAG‐1 stands at the forefront of regenerative dentistry as a master regulator of BMP and Wnt signaling, orchestrating key processes in tooth development and repair. Its inhibition has demonstrated remarkable potential to regenerate functional teeth, restore dental tissues, and address congenital conditions such as tooth agenesis. Our review highlights the unique contribution of single‐cell RNA sequencing in mapping *USAG‐1* expression to specific gingival fibroblast and basal cell populations, offering new insights into the cellular heterogeneity underlying regeneration. Moreover, by elucidating USAG‐1's interactions with LRP5, DKK4, BMP2, BMP4, and BMP7, and exploring its links to SMAD and MAPK/ERK pathways, we reveal its integration within a complex signaling network that extends beyond conventional BMP–Wnt regulation. Importantly, USAG‐1 inhibition appears to differentially influence dentin and pulp regeneration, gingival repair, and alveolar bone remodeling, underscoring its multifaceted therapeutic potential. Despite this promise, critical challenges—including delivery optimization, long‐term safety, regulatory approval, and equitable access—must be addressed before USAG‐1–based therapies become a mainstream clinical solution. As clinical trials advance, the integration of molecular biology, single‐cell transcriptomics, and translational medicine will be essential to fully harness USAG‐1's potential and to pave the way for a paradigm shift in precision regenerative dentistry.

## Author Contributions

Zahra Moradi, Mohammadreza Karimi, Shirin Kolahdouz, Narges Arya, Vahid Akheshteh, Fatemeh Ayanzadeh, Yasmina Aalizadeh, Hanieh Moravvej, Marzie Keshavarzi, and Amar Basri reviewed the literature and collected the data. Zahra Moradi, Mohammadreza Karimi, Shirin Kolahdouz, Narges Arya, Vahid Akheshteh, and Amar Basri analyzed the RNA‐Seq dataset. Zahra Moradi wrote the initial draft and Mohammadreza Karimi, Shirin Kolahdouz, Narges Arya, Vahid Akheshteh, Fatemeh Ayanzadeh, Yasmina Aalizadeh, Hanieh Moravvej, Marzie Keshavarzi, and Amar Basri revised and reviewed the manuscript.

## Funding

The authors received no specific funding for this work.

## Conflicts of Interest

The authors declare no conflicts of interest.

## Supporting information

Table_S1_RNASeq_metrics.

Table_S2.

## Data Availability

The data supporting the findings of this study are derived from publicly available single‐cell RNA sequencing databases, preclinical studies, and clinical trial reports referenced in this review. Specific datasets analyzed during the study are accessible through their respective repositories, as cited in the manuscript. Additional information is available upon request.
